# Heme Oxygenase Contributes to Alleviate Salinity Damage in *Glycine max* L. Leaves

**DOI:** 10.1155/2009/848516

**Published:** 2009-09-29

**Authors:** Carla Giannina Zilli, Diego Mario Santa-Cruz, Gustavo Gabriel Yannarelli, Guillermo Osvaldo Noriega, María Luján Tomaro, Karina Beatriz Balestrasse

**Affiliations:** Departamento de Química Biológica, Facultad de Farmacia y Bioquímica, Universidad de Buenos Aires, Junín 956 (1113), Buenos Aires, Argentina

## Abstract

Plants are frequently subjected to different kinds of stress, such as salinity and, like other organisms, they have evolved strategies for preventing and repairing cellular damage caused by salt stress. *Glycine max* L. plants were subjected to different NaCl concentrations (0–200 mM) for 10 days. Treatments with 100 and 200 mM NaCl induced ion leakage and lipid peroxidation augmentation, loss in chlorophyll content, and accumulation of O_2_^•−^ and H_2_O_2_. However, 50 mM NaCl did not modify these parameters, which remains similar to control values. Catalase, superoxide dismutase, and heme oxygenase (HO-1) activities and gene expressions were increased under 100 mM NaCl, while no differences were observed with respect to controls under 50 mM salt. Treatment with 200 mM NaCl caused a diminution in the enzyme activities and gene expressions. Results here reported let us conclude that HO also plays a leading role in the defense mechanisms against salinity.

## 1. Introduction

 The cultivation of soybean is increasing in the world due to its widespread use in the food and feed industry. However, its yield may be adversely affected by different environmental insults. Abiotic stresses, such as drought, salinity, and extreme temperatures are serious threats to agriculture and the natural status of the environment. As a consequence of global heating, increased salinization of arable land is expected to have devastating effects. Recently, it has been reported that salinity provoked in olive leaves an imbalance between reactive oxygen species (ROS) production and antioxidant defenses, with the induction of oxidative stress [1]. To counteract the toxicity of ROS, defense systems that scavenge cellular ROS have been developed in plants to cope with oxidative stress via the nonenzymatic and enzymatic systems [2]. Enhancement of antioxidant defense in plants can thus increase tolerance to different stress factors. Antioxidants (ROS scavengers) include enzymes such as CAT and SOD, as well as nonenzyme molecules such as ascorbate, glutathione, carotenoids, and anthocyanins.

Among the various genes encoding proteins that possess antioxidant characteristics, there has been a growing interest over the last years in the heme oxygenase system (HO, EC 1.14.99.3), the family of enzymes that control the initial and rate-limiting step in heme catabolism. Heme oxygenase catalyses the oxidation of heme to biliverdin IX*α* (BV), CO, and Fe^2+^. Afterwards, BV is reduced to bilirubin through the action of BV reductase. Both BV and bilirubin have strong antioxidant properties in vitro as well as in vivo [3] and play an important role against oxidative damage [4]. While heme degradation is a common feature of all HOs, this heat shock protein actually has a great variety of roles in the cell, due in part to the diversity of the reaction products. In mammals, it has been demonstrated that one of the three known isoforms, HO-1, is induced by an array of pro-oxidant compounds [5–8]. The release of iron and the subsequent induction of iron-sequestering proteins such as ferritin have also been proposed as an important function of HOs in counteracting oxidative stress [9, 10]. In plants, BV is reduced to phytocochromobilin, the chromophore of the phytochrome family of photoreceptors. Recent reports have demonstrated the presence in soybean leaves and nodules of one HO closely related to the HO-1 of mammalian cells, on the basis of its antioxidant behavior under Cd and UV-B treatment [11, 12].

Salt stress is one of the most important abiotic stresses that adversely affect natural productivity and causes significant crop loss worldwide. Salinity has always been considered a serious constraint on agricultural productivity [13]. Almost every aspect of the plant's physiology and biochemistry is affected. Salt stress is a complex phenomenon that involves morphological and developmental changes. Two major components have been identified in this phenomenon, osmotic stress and ion toxicity [14]. Higher plants have multiple protective mechanisms against salt stress including ion homeostasis, osmolyte biosynthesis, ROS scavenging, water transport, and transducers of long-distance response coordination. It is generally accepted that many stresses, including salinity, induce an overproduction of ROS, such as H_2_O_2_, O_2_^•−^, and hydroxyl radicals, and these species are thought to be responsible for the oxidative damage associated with plant stress. To counteract the toxicity of ROS, defence systems that scavenge cellular ROS have been developed in plants to cope with oxidative stress via the nonenzymatic and enzymatic systems [15, 16].

With the aim of obtaining a better comprehension of the in vivo effect of salinity on oxidative stress defense system, we have analyzed oxidative stress parameters, the behavior of the well-known antioxidant enzymes CAT and SOD, as well as the response of HO-1 to different NaCl concentrations.

## 2. Materials and Methods

### 2.1. Plant Material and Treatments

Surface sterilized *Glycine max.* L. seeds (A6445RG) were germinated directly in plastic pots containing vermiculite in controlled environmental chambers, with a photoperiod of 16 hours, photon flux density of 175 *μ*mol m^−2^ s^−1^, and a day/night regime of 25/20°C, and were simultaneously inoculated with *Bradyrhizobium japonicum* strain E 109 (INTA, Castelar). After this, they were watered daily with a nutrient solution [17] during the first 5 days and then with an N-free nutrient solution. After four weeks, plants were treated with nutrient solution devoid of salt (Control) or containing 50, 100, and 200 mM of NaCl. After 10 days of treatment, leaves were isolated and used for the determinations. When the effect of ZnPPIX was investigated, plants were treated with 22 *μ*M ZnPPIX added to NaCl solutions during 2 days before the end of the experiment.

### 2.2. Ion Leakage Assay

Leaves were harvested and cut into 30 mm pieces. They were washed in deionized water to remove surface added electrolytes and placed in Petri dishes with 15 mL of deionized water at 25°C for 3 hours. Electrical conductivity in the bathing solution was determined (C1). Then, the samples were heated at 80°C for 2 hours and the conductivity was read again in the bathing solution (C2). Relative ion leakage was expressed as a percentage of the total conductivity after heating at 80°C (relative ion leakage % = C1/C2 × 100) [18].

### 2.3. Thiobarbituric Acid Reactive Substances (TBARS) Determination

Lipid peroxidation was measured as the amount of TBARS determined by the thiobarbituric acid (TBA) reaction as described by Heath and Packer [19]. Fresh control and treated leaves (0.3 g) were homogenized in 3 mL of 20% (w/v) trichloroacetic acid (TCA). The homogenate was centrifuged at 3500 × g for 20 minutes. To 1 mL of the aliquot of the supernatant, 1 mL of 20% TCA containing 0.5% (w/v) TBA and 100 *μ*L 4% butylated hydroxytoluene (BHT) in ethanol was added. The mixture was heated at 95°C for 30 minutes and then quickly cooled on ice. The contents were centrifuged at 10 000 × g for 15 minutes and the absorbance was measured at 532 nm. Value for nonspecific absorption at 600 nm was subtracted. The concentration of TBARS was calculated using an extinction coefficient of 155 mM^−1^ cm^−1^.

### 2.4. Chlorophyll Content Determination

Leaves (0.5 g of fresh weight) were homogenized with 96% ethanol (1:30 w/v). Extracts were heated in a boiling bath until complete bleaching. After centrifugation, the absorbance was measured in the supernatant at 665, 649, and 654 nm as described by Wintermans and de Mots [20].

### 2.5. H_2_O_2_ Localization In Vivo

Leaves from control and treated plants were excised and immersed in a 1% solution of 3,3′-diamino benzidine (DAB) in Tris–HCl buffer (pH 6.5), vacuum-infiltrated for 5 minutes, and then incubated at room temperature for 16 hours in the absence of light. Leaves were illuminated until appearance of brown spots characteristic of the reaction of DAB with H_2_O_2_. Leaves were bleached by immersing in boiling ethanol to visualize the brown spots. H_2_O_2_ deposits were determined by scanning spots from leaf pictures and the numbers of pixels were quantified using the public domain NIH Image program (developed at the US National Institutes of Health). The results were expressed as percentage of spots area versus total leaf area ((spot area/total leaf area) × 100) in order to compensate the differences in leaves size [21].

### 2.6. O_2_^•−^ Localization In Vivo

Leaves from control and treated plants were excised and immersed in a 0.1% solution of nitroblue tetrazolium (NBT) in K-phosphate buffer (pH 6.4), containing 10 mM Na-azide, and were vacuum-infiltrated for 5 minutes and illuminated until the appearance of dark spots, characteristic of blue formazan precipitate. Leaves were bleached by immersing in boiling ethanol to visualize the dark spots. Superoxide deposits were quantified by scanning spots from leaf pictures as mentioned earlier [21].

### 2.7. Antioxidant Enzymes Preparations and Assays

Extracts for determination of catalase (CAT, EC 1.11.1.6) and superoxide dismutase (SOD, EC 1.15.1.1) activities were prepared from 0.3 g of leaves homogenized under ice-cold conditions in 3 mL of extraction buffer, containing 50 mM phosphate buffer (pH 7.4), 1 mM EDTA, 1 g PVP, and 0.5% (v/v) Triton X-100 at 4°C. The homogenates were centrifuged at 10 000 × g for 20 minutes and the supernatant fraction was used for the assays. CAT activity was determined in the homogenates by measuring the decrease in absorption at 240 nm in a reaction medium containing 50 mM potassium phosphate buffer (pH 7.2) and 2 mM H_2_O_2_. The pseudo-first-order reaction constant (*k*′ = *k*. [CAT]) of the decrease in H_2_O_2_ absorption was determined and the CAT content in pmol mg^−1^ protein was calculated using *k* = 4.7 × 10^7^ M^−1^s^−1^ ) [22]. Total SOD activity was assayed by the inhibition of the photochemical reduction of NBT, as described by Becana et al. [23]. The reaction mixture consisted of 50–150 *μ*L of enzyme extract and 3.5 mL O_2_^•−^ generating solution which contained 14.3 mM methionine, 82.5 *μ*M NBT, and 2.2 *μ*M riboflavin. Extracts were brought to a final volume of 0.3 mL with 50 mM K-phosphate (pH 7.8) and 0.1 mM Na_2_EDTA. Test tubes were shaken and placed 30 cm from light bank consisting of six 15-W fluorescent lamps. The reaction was allowed to run for 10 minutes and stopped by switching the light off. The reduction in NBT was followed by reading absorbance at 560 nm. Blanks and controls were run the same way but without illumination and enzyme, respectively. One unit of SOD was defined as amount of enzyme which produced a 50% inhibition of NBT reduction under the assay conditions.

### 2.8. Heme Oxygenase Preparation and Assay

Leaves (0.3 g) were homogenized in a Potter-Elvehejm homogenizer using 1.2 mL of ice-cold 0.25 M sucrose solution containing 1 mM phenylmethyl sulfonyl fluoride, 0.2 mM EDTA, and 50 mM potassium phosphate buffer (pH 7.4). Homogenates were centrifuged at 20 000 × g for 20 minutes and chloroplasts were used for activity determination. Heme oxygenase activity was assayed as previously described with minor modifications [24]. The assays (1 mL final volume) contained 250 *μ*L of extract (0.5 mg protein), 10 *μ*M hemin, 0.15 mg mL^−1^ bovine serum albumin, 50 *μ*g mL^−1^ (4.2 *μ*M) spinach (Spinacia oleracea) ferredoxin (Sigma Chemical Co.), 0.025 units mL^−1^ spinach ferredoxin-NADP^+^ reductase (Sigma Chemical Co.). The reaction was started by adding NADPH to a final concentration of 100 *μ*M, samples were incubated at 37°C for 30 minutes and BV formation was calculated using the absorbance change at 650 nm. The concentration of BV was estimated using a molar absorption coefficient at 650 nm of 6.25 mM^−1^ cm^−1^ in 0.1 M HEPES-NaOH buffer (pH 7.2). One unit of the enzyme forms 1 nmol of biliverdin in 30 minutes under assay conditions.

### 2.9. Western-Blots Analysis for HO

Homogenates obtained for HO activity assays were also analyzed by Western immunoblot technique. Forty *μ*g of protein from leaf homogenates were subjected to sodium dodecyl sulfate (SDS)-poliacrylamide gel electrophoresis using a 12% acrylamide resolving gel (Mini Protean II System, BioRad, Hertz, UK), according to Laemmli [25]. Separated proteins were then transferred to nitrocellulose membranes and nonspecific binding of antibodies was blocked with 3% nonfat dried milk in PBS, pH 7.4 for 1 hour at room temperature. Membranes were then incubated overnight at 4°C with primary antibodies raised against *Arabidopsis thaliana* HO-1, [26] diluted 1:2000 in Tris-NaCl buffer plus 1% nonfat milk. Immune complexes were detected using peroxidase-conjugated goat antirabbit inmunoglobulin G and visualized with the enhanced chemiluminescence western blotting procedure (ECL, Amersham Pharmacia Biotech, Uppsala, Sweden). The films were scanned (Fotodyne Incorporated, Wiss, USA) and analyzed using Gel-Pro Analyzer 3.1 software (Media Cybernetics, Md, USA).

### 2.10. Isolation of RNA and RT-PCR Analysis

Total RNA was extracted from soybean leaves by use of Trizol reagent (Gibco BRL). Four micrograms of total RNA were treated with RNase-free DNase I (Promega, Calif, USA) and then 1.0 *μ*g was reverse transcribed into cDNA using random hexamers and M-MLV Superscript II RT (Invitrogen, Calif, USA). PCR reactions were carried out using *Glycine max* HO-1 and 18S specific primers, as previously described [12]. In addition, CAT gene expression was assessed by use of a primer pair specific to *Glycine max* CAT cDNA (sense primer, 5′-CTGCTGGAAACTATCCTGAGTG-3′; antisense primer, 5′-ATTGACCTCTTCATCCCTGTG-3′). The PCR profile was set at 94°C for 1 minute and then 29 cycles at 94°C for 0.5 minute, 54°C for 1minute, and 72°C for 1 minute, with a final extension at 72°C for 7 minutes. Primers 5′-TTCCGAATTCAAAGGTCCAG-3′ and 5′-TAAGATCAGCCACCCTCAGC-3′ were designed to amplify the *Glycine max* SOD cDNA. Cycling conditions were as follows: 94°C for 1 minute, then 33 cycles at 94°C denaturing for 0.5 minute, 55°C annealing for 1 minute, and 72°C extension for 1 minute, and then a final step of 72°C for 7 minutes. Each primer set was amplified using an optimized number of PCR cycles to ensure the linearity requirement for semiquantitative RT-PCR analysis. The amplified transcripts were visualized on 1.5% agarose gels with the use of ethidium bromide. Gels were then scanned (Fotodyne Incorporated, Wiss, USA) and analyzed using Gel-Pro Analyzer 3.1 software (Media Cybernetics, Md, USA).

### 2.11. Protein Determination

Protein concentration was evaluated by the method of Bradford [27] using bovine serum albumin as a standard.

### 2.12. Statistical Analysis

All treatments were repeated four times, with newly grown plants. Data in the text and tables indicate mean values ± SD. Differences among treatments were analyzed by one-way ANOVA, taking *P* < .05 as significant according to Turkey's multiple range test.

## 3. Results

### 3.1. Evaluation of Salt Toxicity

Oxidative stress is the result of excessive production of oxidant species and/or depletion of intracellular antioxidant defenses, leading to an imbalance in the redox status of the cell. Reactive oxygen species are regarded as initiators of peroxidative cell damage. Therefore, TBARS formation in plants exposed to adverse environmental conditions is a reliable indicator of tissue free radical formation. As shown in Table 1, a 22 and 37% increase occurred in plants treated with 100 and 200 mM NaCl, resp., whereas no difference respect to controls were observed in 50 NaCl-treated plants.

Loss of membrane integrity during stress was studied by measuring the leakage of electrolytes from soybean leaves (Table 1). Salinity stress induced a maximum relative injury (192%), when soybean plants were subjected to 200 mM NaCl and 53% with 100 mM NaCl, whereas plants treated with 50 mM NaCl revealed no differences with respect to controls.

Reduction of chlorophyll content can be used as a marker to measure the effect of salinity stress on the integrity of chloroplasts. Table 1 shows that this parameter decreased under 100 and 200 mM NaCl treatment (24 and 58%, resp.), while plants subjected to 50 mM did not differ from controls.

### 3.2. H_2_O_2_ and **O**_2_^•−^ Localization In Vivo

**Figure 1:**
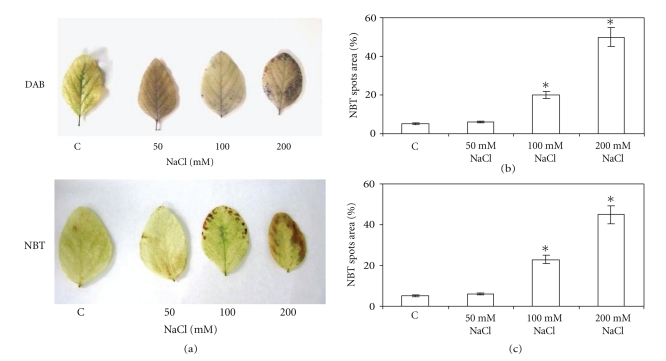
Histochemical detection of H_2_O_2_ and O_2_^•−^ in soybean leaves treated with different NaCl concentrations. The second pair of fully expanded leaves upper the cotyledons were used for the assays as described in Experimental section. (a) Leaves from control and NaCl-treated plants stained for H_2_O_2_ (DAB) and O_2_^•−^ (NBT) content. The figure is representative of four different experiments. Hydrogen peroxide (b) and superoxide anion deposits (c) were quantified by measuring the number of pixels of spots using the NIH Image program (National Institutes of Health, USA). Results are expressed as percentage of spot area versus total leaf area. *P < .05 compared to control, according to Tukey's multiple range test.

In order to assess the oxidative damage caused by salt stress, accumulation of H_2_O_2_ and O_2_^•−^ were performed in vivo by histochemical methods (Figure 1(a)). As shown in Figure 1(b), (c), treatment with 100 mM NaCl produced 15% and 18% H_2_O_2_ and O_2_^•−^ spots area versus total leaf area, respectively, while 200 mM salt treatment markedly increased both reactive species oxygen (45% for H_2_O_2_ and 40% for O_2_^•−^). The lower NaCl concentration (50 mM) did not produce neither H_2_O_2_ nor O_2_^•−^ in soybean leaves (Figure 1).

### 3.3. Changes in Classical Antioxidant Enzymes in Response to Salinity

**Figure 2:**
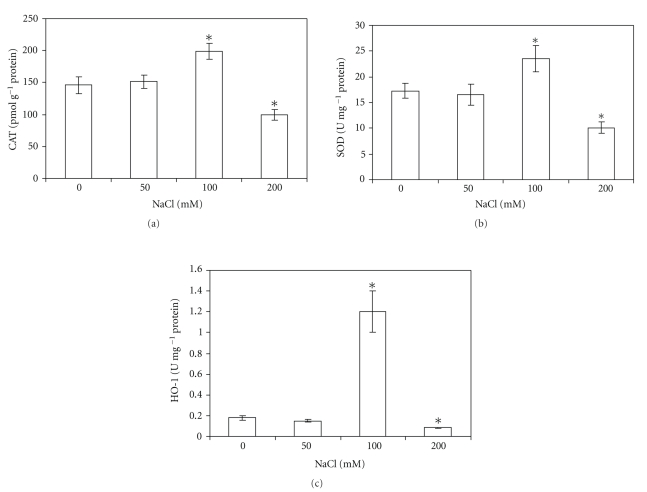
Effect of different salt concentrations on classical antioxidant and heme oxygenase enzyme activities: (a) catalase, (b) superoxide dismutase, and (c) heme oxygenase. Enzymes activities were measured as described in Experimental section. Values are the means of four different experiments (*n* = 4), and bars indicate SD, *significant differences (*P* < .05) compared to control, according to Tukey's multiple range test.

In order to get further insight into the effect all salt-stress on oxidative stress parameters, CAT and SOD activities and gene expression were determined. As shown in Figure 2(a), 100 mM NaCl caused a 36% enhancement of CAT activity respect to controls. On the other hand, under 50 mM NaCl, there was no increase in CAT activity respect to controls, but a significant inhibition (32%) was observed in the presence of 200 mM NaCl. RT-PCR analysis revealed that these activities are positively correlated with gene expression (Figure 4). SOD activity (Figure 2(b)) and gene expression (Figure 4) were increased under 100 mM NaCl (30%), and no differences were observed respect to controls under 50 mM salt. Treatment with 200 mM NaCl caused a diminution in enzyme activity (35%) as well as in gene expression (20%).

### 3.4. Heme Oxygenase Response to Salinity

**Figure 3:**
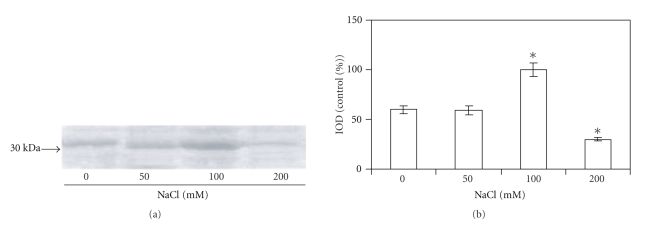
HO-1 protein expression in soybean leaves exposed to different NaCl concentrations (50, 100, or 200 mM) during 10 days. (a) HO-1 protein expression was analyzed by Western blotting as described in Section 2. (b) Densitometry was done by Gel-Pro analyzer to quantify HO-1 protein expression Values are the mean of four independent experiments and bars indicate SD, *significant differences (*P* < .05) compared to control, according to Tukey's multiple range test.

**Figure 4:**
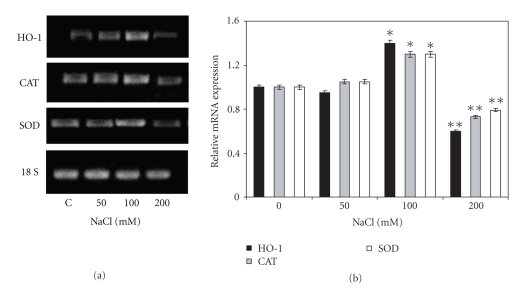
HO-1, CAT, and SOD gene expression in soybean leaves subjected to different NaCl concentrations (50, 100, or 200 mM) during 10 days. (a) HO-1, CAT, and SOD mRNA expression was analyzed by semiquantitative RT-PCR as described in Section 2. The 18S amplification band is shown to confirm equal loading of RNA and RT efficiency. (b) Relative HO-1, CAT, and SOD transcript expressions. Values are the mean of four independent experiments and bars indicate SD, *P < .01, ^**^ *P* < .001 with respect to control according to Tukey's multiple range test.

As shown in Figure 2(c), under 50 mM NaCl there was no increase in the HO activity respect to controls. Concentration of 100 mM NaCl was capable of inducing HO-1 activity (7 fold), while 200 mM salt produced a decrease in the enzyme activity. When protein amount (Figure 3) and gene expression (Figure 4) were assayed, a positive correlation with enzyme activity was found.

### 3.5. Effect of Zn-Protoporphyrin IX on Oxidative Stress Parameters and HO Activity

To determine the involvement of HO-1 in the defense system against oxidative damage caused by salinity, the effect of ZnPPIX, a well-known HO-1 strong inhibitor, on TBARS, ion leakage, chlorophyll content, and HO activity was assayed. As expected, ZnPPIX caused a decreased in enzyme activity (Table 2). Simultaneous treatment with 100 mM NaCl and ZnPPIX significantly enhanced TBARS levels (49%) and ion leakage (130%) when compared with the values obtained when only 100 mM NaCl was added. However, the diminution produced in the chlorophyll content by this saline concentration was not altered by ZnPPIX addition, and therefore, remained similar to that found with 100 mM salt alone. Treatment with ZnPPIX alone did not modify ion leakage, TBARS levels, and chlorophyll content, compared to controls.

## 4. Discussion

This study was undertaken to evaluate in soybean leaves, the in vivo response of HO-1 to salinity. Salt stress disrupts plant ion homeostasis, resulting in excess toxic Na^+^ in the cytoplasm and a deficiency of essential ions such as K^+^ [28]. Drought, salinity, and extreme temperatures are often interconnected, and may induce similar cellular damage by means of ROS formation [29, 30]. For example, drought and/or salinization are manifested primarily as osmotic stress, resulting in the disruption of homeostasis and ion distribution in the cell [31, 32]. Oxidative stress, which frequently accompanies high temperature, salinity, or drought stress, may cause denaturing of functional and structural proteins [33]. As a consequence, these diverse environmental stresses often activate similar cell signaling pathways [33–36] and cellular responses, such as the production of stress proteins and upregulation of antioxidants [37, 38]. In order to establish whether oxidative stress occurred under our experimental conditions, TBARS levels, ion leakage, and chlorophyll content were determined. Katsuhara et al. [39] demonstrated that peroxidation of membrane lipids is an indicator of membrane damage under salt stress. From our experiments, it can be concluded that 200 mM NaCl is effective in triggering membrane damage and leakage. On the other hand, chlorophyll content is diminished under this salt concentration. This result is in agreement with those from Kahn [40] and Abdelkader et al. [41] that postulated that plants grown either under conditions of increased salinity or treated with salt solutions have a reduced chlorophyll accumulation.

To mount an appropriate oxidative stress defense, cells must develop oxidative stress sensors. A number of antioxidant responses have been studied in bacteria, and it has been established that ROS are directly sensed by key regulatory molecules that activate the expression of genes encoding antioxidant proteins at transcription level [42, 43]. Superoxide dismutase, which catalyses the dismutation of O_2_^•−^ to O_2_ and H_2_O_2_, is one of the major cellular defense enzymes that perform a vital role in protecting cells against the toxic effects of O_2_^•−^. Catalase converts harmful metabolic byproducts and, under normal physiological conditions, it controls the H_2_O_2_ concentration so that it does not reach toxic levels that could bring about oxidative damage inside peroxisomes and the surrounding cytoplasm [44]. The results presented in this study showed that soybean leaves SOD gene was induced by 100 mM NaCl, no induction was observed under 50 mM, and a downregulation occurred in plants treated with 200 mM NaCl. It was also found that these gene expressions were positively correlated with enzyme activity. The same behavior was observed when CAT gene expression and activity were analyzed. According to our results, it can be suggested that under 50 mM NaCl the antioxidant defense system can cope with ROS damage, under 100 mM NaCl SOD and CAT are induced in an attempt to protect the cell against this insult, and under 200 mM NaCl ROS production overwhelms the antioxidant defense system of the cell.

The oxidative stress response and its relationship to heat shock phenomena are being intensely investigated in plant tissues [45, 46]. To survive under environmental stress conditions, plants undergo a process of stress acclimation, which may require changes in the flow of metabolites, suppression of pathways involved in the excessive production of ROS, and the induction of various defense genes such as heat shock proteins and ROS scavenging enzymes [30, 47, 48]. The downregulation of SOD and CAT genes as well as the inhibition of enzyme activities could be attributed to ROS-induced alterations, such as transductional modifications, DNA damage, protein fragmentation, and increased susceptibility to proteolysis and cross-linking reactions [33]. These results are in agreement with the observed H_2_O_2_ and O_2_^•−^ localization in situ. The involvement of H_2_O_2_ as a leading ROS in Cd-induced oxidative stress generation has been reported in tobacco cell cultures [49] and both H_2_O_2_ and O_2_^•−^ have been shown to act directly or indirectly in signal transduction of defense responses [50]. Studies carried out in rat liver suggested that HO-1 induction occurs when steady-state levels of the ROS are increased and defenses (e.g., CAT, SOD) are decreased [51]. To the best of our knowledge, no information is still available on the behavior of HO-1 of plant tissues subjected to salinity. If we compare the expression profile of CAT, SOD, and HO, it is clear that these enzymes respond to the same signal in the same direction. Liberation of Fe, a potent oxidant that may exacerbate oxidative stress [52], takes place as a consequence of heme degradation. It is interesting to note that ferritins play an essential role in iron homeostasis by sequestering iron in a bioavailable and nontoxic form. In plants, ferritin mRNAs are highly and quickly accumulated in response to iron overload. Such accumulation leads to a subsequent ferritin protein synthesis and Fe storage [53].

During the last five years, it has been established that the HO-1 induction could alleviate the oxidative damage caused by Cd or UV-B radiation in soybean plants [11, 12]. When plants were cotreated with ZnPPIX, an enhancement of ROS production was observed. The effect elicited by ZnPPIX was not due to a pro-oxidant action because, when it was employed alone, the parameters associated with the oxidative stress, TBARS levels, ion leakage, and chlorophyll content, were not modified. However, these results imply that the pro-oxidant effect of ZnPPIX when it was administered together with 100 mM NaCl was due to the lack of HO-1 activity resulting from the presence of the inhibitor, indicating the role that this heat-shock enzyme plays in the attenuating salinity-induced damage.

Taken together, the present results allow us to conclude that under salinity HO-1 gene expression, protein amount and activity are enhanced in an attempt to protect leaves tissues against this insult. Nevertheless, when the increase in H_2_O_2_ and O_2_^•−^ formation overwhelms the defense capacity of the cell, HO-1 gene is downregulated as it occurs with SOD and CAT. Inhibition of HO-1 activity demonstrated that this enzyme plays a leading role in the defense mechanism against salinity, and it could be considered as essential component of enzymatic antioxidant defense system in plant tissues.

Further studies on the comparison of antioxidant enzyme profiles between salinity-tolerant and sensitive genotypes are necessary to determine if, like many newly discovered genes, HO may serve as a candidate for modifying plant salinity tolerance.

## Figures and Tables

**Table 1:** Effect of different salt concentrations on TBARS levels, ion leakage and chlorophyll content.

Treatment	TBARS (nmol g^−1^ FW)	Relative ion leakage (%)	Chlorophyll (a+b) (mg g^−1^ FW)
Control	40.17 ± 1.33^(a)^	100 ± 6^(a)^	3.35 ± 0.40^(a)^
50 mM NaCl	40.87 ± 0.50^(a)^	111 ± 10^(a)^	3.20 ± 0.40^(a)^
100 mM NaCl	49.08 ± 1.50^(b)^	153 ± 10^(b)^	2.54 ± 0.30^(b)^
200 mM NaCl	55.12 ± 1.00^(c)^	292 ± 20^(c)^	1.40 ± 0.13^(c)^

Data are mean values of four independent experiments ± SD. Different letters within columns indicate significant differences (*P* < .05) according to Tukey's multiple range test.

**Table 2:** Effect of ZnPPIX on HO-1 activity, TBARS levels, ion leakage, and chlorophyll content in soybean leaves subjected to salt stress.

Treatment	HO-1 (U mg^−1^ protein)	TBARS (nmol g^−1^ FW)	Relative ion leakage (%)	Chlorophyll (a+b) (mg g^−1^ FW)
Control	0,135 ± 0.031^(a)^	40.17 ± 1.33^(a)^	100 ± 6^(a)^	3.35 ± 0.40^(a)^
100 mM NaCl	1,210 ± 0.223^(b)^	49.08 ± 1.50^(b)^	153 ± 10^(b)^	2.54 ± 0.30^(b)^
ZnPPIX	0.059 ± 0.004^(c)^	38.23 ± 2.10^(a)^	103 ± 13^(a)^	3.01 ± 0.27^(a)^
ZnPPIX + 100 mM NaCl	0.063 ± 0.006^(c)^	59.76 ± 4.11^(c)^	230 ± 15^(c)^	1.41 ± 0.23^(c)^

Data are mean values of four independent experiments ± SD. Different letters within columns indicate significant differences (*P* < .05) according to Tukey's multiple range test.
