# Correction to “Heme Oxygenase Contributes to Alleviate Salinity Damage in *Glycine max* L. Leaves”

**DOI:** 10.1155/ijcb/9785268

**Published:** 2026-08-24

C. G. Zilli, D. M. Santa‐Cruz, G. G. Yannarelli, G. O. Noriega, M. L. Tomaro, and K.B. Balestrasse, “Heme Oxygenase Contributes to Alleviate Salinity Damage in *Glycine max L.* Leaves,” *International Journal of Cell Biology* 2009, no. 1 (2009): 1–9, https://doi.org/10.1155/2009/848516.

In the article titled “Heme Oxygenase Contributes to Alleviate Salinity Damage in *Glycine max L*. Leaves,” there were labeling errors in Figure 1. Specifically, the labels indicating the stains (NBT or DAB) for both sets of leaf images were swapped in error within Figure 1a, whereas the chart presented in Figure 1c was incorrectly labelled as NBT. These errors occurred during figure assembly and Figure 1 should be corrected as follows:

**Figure 1:**
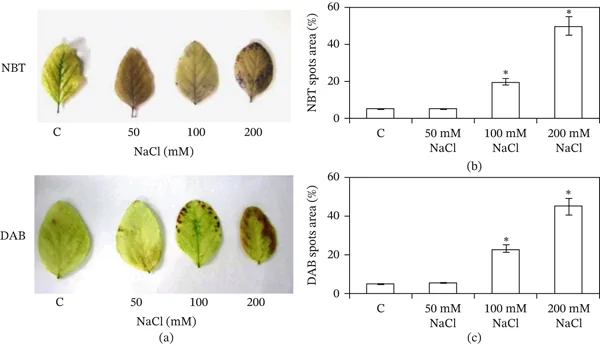
Histochemical detection of H_2_O_2_ and O_2_^−^ in soybean leaves treated with different NaCl concentrations. The second pair of fully expanded leaves upper the cotyledons were used for the assays as described in the Experimental section. (a) Leaves from control and NaCl‐treated plants stained for H_2_O_2_ (DAB) and O_2_^−^ (NBT) content. The figure is representative of four different experiments. (b) Hydrogen peroxide and (c) superoxide anion deposits were quantified by measuring the number of pixels of spots using the NIH (National Institutes of Health) image program (United States). Results are expressed as percentage of spot area versus total leaf area.  ^∗^ *p* < 0.05 compared with control, according to Tukey′s multiple range test.

We apologize for this error.

